# Validation of a quality-of-life measure for older people using urgent and emergency care

**DOI:** 10.1186/s12955-025-02454-z

**Published:** 2025-11-28

**Authors:** Kiri Lay, Julie Ratcliffe, Rachel Milte, Diana Khanna, Craig Whitehead, Jyoti Khadka

**Affiliations:** 1 Health and Social Care Economics Group, Caring Futures Institute, Flinders University, GPO Box 2100, Adelaide, SA 5001 Australia; 2 Rehabilitation, Aged and Palliative Care, Southern Adelaide Local Health Network, Adelaide, SA Australia

**Keywords:** Construct validity, Urgent care, Quality-of-life, Older people

## Abstract

**Background:**

Older people (aged 65 years and over) frequently present to urgent or emergency care settings, often with multiple health and social care needs. Despite this, their quality-of-life (QOL) is rarely assessed in a systematic or meaningful way. Capturing QOL in these settings is essential for delivering person-centred care for guiding improvements in service planning and delivery. This study aimed to evaluate the construct validity of the Quality-of-life – Aged Care Consumers, (QOL-ACC), an older people specific QOL instrument, in the context of urgent or emergency care.

**Methods:**

Data were collected via an online survey which included demographic questions, the QOL-ACC, the EQ–5D-5L (a health-specific measure), the Urgent Care Questionnaire (UCSQ) and global self-reported health and QOL questions. Construct validity was assessed through Convergent and known-group validity. Convergent validity was assessed using 13 a priori hypotheses predicting correlations between the QOL-ACC and its dimensions and the other validated instruments. Known group validity was assessed with four a priori hypotheses comparing QOL-ACC scores across subgroups defined by self-rated health and QOL, areas of socio-economic advantage and disadvantage care needs at home.

**Results:**

Among 205 respondents (mean age 75 ± 6.0 years, 59% female), 37 (18.0%) were receiving home-based aged care services. The QOL-ACC utility scores demonstrated moderate correlation with the EQ-5D-5L (ρ = 0.60) and the EQVAS (ρ = 0.57). Low to moderate correlation was demonstrated with the 3 dimensions of the UCSQ (ρ = 0.27 ρ = 0.34, ρ = 0.37). The QOL-ACC was able to discriminate between groups with different self-rated health and QOL levels (*P* < 0.001). No significant differences were observed by areas of socio-economic advantage and disadvantage nor by care needs at home.

**Conclusions:**

The QOL-ACC demonstrated strong construct validity for assessing QOL in older people accessing urgent or emergency care. Its ability to distinguish between self-rated health and QOL, with consistent scores across socio-economic groups and care use, supports its broad applicability. Further research is needed to assess its reliability and responsiveness in these settings.

**Supplementary Information:**

The online version contains supplementary material available at 10.1186/s12955-025-02454-z.

## Background

Pressure on emergency departments throughout the developed world is increasing and many countries face major challenges to continue to provide effective and efficient emergency medical services [1, 2]. In Australia, where the provision of emergency medicine is a vital element of the health system, hospital emergency departments are frequently overwhelmed, with only 64% of patients, triaged as emergency, seen within target wait times across Australia in 2022-23 [3]. Older people (aged 65 years and over) are more likely to access health services, including emergency departments accounting for 24% of emergency department presentations in Australia despite representing only 17% of the population [3]. Among older people, men and those aged 85 years and over attend emergency departments more frequently than women and those aged 65–84 years [3]. Moreover, older people accessing aged care services at home attend emergency departments at a higher rate than those accessing residential aged care services [4]. Approximately 50% of older people seen in emergency departments are subsequently admitted to hospital compared to 29% of all patients [3]. Urgent care facilities have been funded by the Australian government since 2022-23 in a bid to relieve pressure on hospital emergency departments [5]. In Australia, urgent care centres are extended General Practitioner clinics which are equipped to provide diagnostic and assessment services. They are designed to deliver care for acute non-life-threatening episodic illness that do not require immediate attention at a hospital. Australia has a universal health care system where primary care (such as at a general practice clinic) increasingly requires co-contribution whereas hospital care, particularly emergency care is largely ‘bulk-billed’ and therefore fee-free. Urgent care clinics are also ‘bulk-billed’ for all patients thus providing fee-free services [5].

The routine collection of patient perspectives in urgent care and other clinical services through the embedded use of Patient Reported Measures (PRMs) facilitates the inclusion of the patients’ voices and can help to improve patient experience as well as identify areas for improvement in the health service [6, 7]. There are also calls for a standardised outcome measurement protocol to enable direct comparison and data sharing and comparison across health services both within Australia and internationally, however, the routine collection of QOL data through PRMs is not yet consistent across the sector [6]. In Australia, where health care funding is shared between Federal and State Governments, standards and guidelines around the collection of health outcome data tend to vary between jurisdictions [2].

The routine measurement of health outcomes in Australia’s health system is strongly recommended by the Australian Commission on Safety and Quality in Health Care and is promoted in the 2020-25 National Health Reform Agreement [6, 8]. Guidelines published by the Australian Commission on Safety and Quality in Health Care recommend the routine collection of PRM data and the selection of an outcome measure that captures outcomes that are important to the patient and is appropriate for the patient population, that is, easy to complete and without burden. They also recommend that outcome measurement avoid duplication, for instance, where both a disease-specific and generic outcome measure are selected [8]. Whilst these guidelines provide these broad recommendations on the selection of PRMs for outcome measurement, there are no explicit requirements for PRM outcome measurement in clinical settings currently operational [9].

Due to the frequent transition of older people across health and care services, the routine collection of patient-reported outcome data for older people requires consistent measurement across both sectors [10]. Tracking of outcomes even across a single incident may include interaction with multiple health and care services. The EQ-5D is the most commonly used preference-based PRM in health care settings and is designed to measure health-related QOL (HRQOL). However, many interventions for older people aim to achieve broader QOL outcomes, such as enabling or prolonging independent living [11]. There is a strong body of evidence to suggest that older people value health and QOL differently to younger people, rating dimensions such as independence, autonomy and physical mobility relatively higher [12]. Additionally, there is an increasing policy shift in Australia towards the integration of health and care for older people in recognition of their unique needs and in attempts to alleviate some of the burden on the health system [13]. Therefore, it is essential that QOL measures are used as relevant and appropriate for older people across settings.

The Quality-of-Life Aged Care Consumers (QOL-ACC) is an older person-specific measure that was developed in consultation with aged care services users to measure QOL in an aged care context [14–16]. It has been extensively validated with older individuals receiving both in-home and residential aged care services, proving to be a valid and reliable tool for this population [17–21]. In 2023, the QOL-ACC was adopted as part of the National Quality Indicators Program following recommendations from the 2019 Royal Commission into Aged Care Quality and Safety [22]. A preference based scoring algorithm representative of an older Australian population was developed for the QOL-ACC in 2023 which facilitates the generation of utility scores, in turn enabling the generation of Quality Adjusted Life Years (QALYs) which are the typical unit of outcome measurement for cost-utility analysis [23]. Cost-utility analysis is the most commonly utilised method of economic analysis for health funding allocation decisions in Australia and is recommended by the Australian Pharmaceutical Benefits Advisory Committee [24]. Building on its adoption in the National Aged Care Quality Indicator Program, extending use of the QOL-ACC into urgent care would help to align measurement across health and aged care, supporting system integration and more person-centred service delivery.

While other PRMs have been developed with the aim of assessing the QOL of older people, such as the Adult Social Care Outcomes Toolkit (ASCOT) [25] and Investigating Choice Experiments Capability Measure for Older People **(**ICECAP-0) [26] measures, the QOL-ACC measures is distinctive in encapsulating the domains identified by older people themselves as most important to their QOL, while being suitable for use in economic evaluation for health and social care interventions. Additionally, given its demonstrated validity and reliability in long-term aged care settings, the QOL-ACC is likely to be suitable for measuring QOL among community dwelling older people who frequently interact with health services and for tracking patient-reported outcomes across settings. Facilitating the use of the QOL-ACC for collection of aggregated data in this context could inform service evaluation and benchmarking, enabling urgent care providers to monitor the quality of outcomes for older people beyond traditional clinical indicators. However, the QOL-ACC has not, thus far, been validated for older people in general health services contexts or those accessing urgent or emergency care. Validating the QOL-ACC in this new context, will help assess it feasibility and construct validity for older people in urgent or emergency care settings, and potentially extends its utility as a standardised tool for broader health services evaluation.

## Methods

### Study sample and recruitment

Participants were recruited across all Australian states and territories through the Pureprofile online panel in January and February 2024. Inclusion criteria included those aged 65+, living in the community or in aged care and had accessed an emergency or urgent care clinic at least once in the previous 12 months. Registered individuals who met the criteria were forwarded the link to the online survey completion platform containing all survey tools. An additional screening question regarding urgent or emergency care visits was included on the platform to ensure that all respondents met the selection criteria. The survey tools included the QOL-ACC, the EQ-5D-5L and a validated quality-of-care experience measure, the Urgent Care Service Questionnaire (UCSQ) (all three measures are described in further detail below). In addition, participants were asked questions about their urgent/emergency care clinic visit, any other/further visits, follow up care, as well as demographic questions regarding age, living situation, education, support needs, language and country of birth. All participants provided informed consent and ethical approval was provided by Flinders University Social and Behavioural Research Ethics Committee (Project number 6358).

### Instruments

#### QOL-ACC (Quality-of-Life-Aged Care Consumers)

The QOL-ACC is a self-reported outcome measure focused on health and wellbeing aspects of QOL, specifically developed for use with older people in an Australian aged-care context. The QOL-ACC contains six dimensions, Mobility, Emotional Wellbeing, Pain Management, Social Connection, Activity and Independence. Response options are on a 5-point scale (All of the time - None of the time) and the reference period is ‘today’. QOL-ACC utilities were calculated for this study using the scoring algorithm based on Australian older population derived preferences developed by Ratcliffe et al. [23].

#### EQ-5D-5L

The EQ-5D was developed by the EuroQoL group and is a HRQOL outcome measure comprising five dimensions plus a Visual Analogue Scale (EQ-VAS). The EQ-5D is a generic HRQOL measure and is designed to be used to assess the HRQOL of a variety of populations and in various health and care contexts. The EQ-5D-5L has a five level Likert type scale response option, with responses ranging from ‘No Problems’ to ‘Extreme problems/Unable’. The EQ-VAS asks the respondent to rate their health on a scale from 0-100 where 0 represents the worst and 100 the best health imaginable [27]. For this study the preference-based scoring algorithm developed by Norman et al. based on Australian population preferences was used [28].

#### UCSQ

The UCSQ is a quality-of-care experience measure developed for the assessment of care experience specifically in urgent care contexts to facilitate measurement of urgent and emergency care services from the patient perspective [29]. Developed through qualitative investigations with recent urgent and emergency care users, it consists of three dimensions, Entry, Convenience and Progress with a total of 22 questions [30]. Responses are given on a 5-point scale from ‘strongly agree’ through ‘strongly disagree. Each dimension is scored separately utilising a summary scoring method with possible scores ranging from 0–13 for Progress, 0–4 for Entry and 0–5 for patient convenience where higher scores represent better care experience.

### Construct validity

Validity testing for this study was based on guidelines from the Consensus-based Standards for the Selection of Health Measurement Instruments (COSMIN) [31]. Construct validity refers to the extent to which a survey tool, or items within the tool, measure the construct they are designed to measure [32]. For this study, we assessed construct validity through convergent and known group validity with hypothesis testing. Convergent validity is assessed by testing the associations between the measure and the items within the measure with other similar validated survey tools or global questions against a list of hypotheses developed a-priori. Known group validity is assessed by testing the discriminatory power of the measure in sample groups with expected, hypothesised differences. An initial list of 24 potential hypotheses was developed by the first author (KL) following a literature search of previous construct validity studies, including those for the QOL-ACC in aged care. This list was distributed to co-authors who provided written feedback and an additional two suggested hypotheses after which the first (KL) and last author (JK) discussed the feedback and revised the list. A total of 17 hypotheses were developed a-priori through this process, referenced literature is cited in the hypothesis table. Convergent validity was tested by assessing correlation between the QOL-ACC and its items and two existing validated measures, the UCSQ and the EQ-5D-5L. The strength and direction of the correlation was hypothesised. For example, we hypothesised that the QOL-ACC would show a positive, moderate correlation with the global self-rated QOL score and the global self-rated health score. For the UCSQ, the Entry and Convenience dimensions were hypothesised to be less correlated with ongoing QOL than the Progress dimension. Whilst the QOL-ACC dimensions are different in key ways to the EQ-5D-5L dimensions some were nevertheless predicted to be correlated with their closest QOL-ACC equivalent, for example the ‘Mobility’ and ‘Pain’ dimensions. See Table 1 for a list of all hypotheses. A low to medium significant correlation (ρ >0.30 and ρ < 0.70) is considered sufficient to affirm good convergent validity with higher correlation expected for more similar constructs [33]. Based on published guidelines convergent validity was deemed sufficient if the analysis confirmed more than 75% of the expected relationships in terms of both direction and strength of correlations [33, 34].

**Table 1:** A priori hypothesised association between the Quality-of-Life Aged Care Consumer (QOL-ACC) instrument, it’s dimensions and other related constructs – Convergent validity

Hypothesis No.	Instrument/Dimension	Expected relationships with the QOL-ACC and its dimensions	Achieved
Convergent Validity (1413)			
QOL-ACC utility scores and other instruments			
1	EQ-5D-5L	As the EQ-5D-5L measures Health Related Quality-of-life, a construct closely related to Quality-of-life and as the QOL_ACC and the EQ-5D-5L contain two similar dimensions a low to moderate correlation was expected between QOL-ACC sand EQ-5D-5L utility scores	Yes
2	EQ-VAS	Good health is indicative of better quality-of-life and therefore scores on the EQ-VAS were expected to display low to moderate positive correlation with QOL-ACC utility scores [29].	Yes
	UCSQ	Quality of Life and Quality of Care experience are related but separate constructs, previously the QOL-ACC has been found to correlate with a different care experience measure (the QCE-ACC) and as such a positive correlation is expected between the QOL-ACC utility score and the UCSQ scores [14].	
3	UCSQ – Entry	Positive low correlation expected as above.	**No**
4	UCSQ - Convenience	Positive low correlation expected as above.	Yes
5	UCSQ - Progress	As the Progress score is more indicative of ongoing care experience and therefore QOL at the time of the survey a low to moderate correlation is expected.	Yes
QOL-ACC dimensions			
6	EQ-5D-5L - Mobility	QOL-ACC Getting around dimension and EQ-5D-5L Mobility dimension are measuring the same concept of getting around, however the QOL-ACC allows specifically for the use of mobility aids, hence, a positive and low-moderate correlation between scores on these two dimensions are expected.	Yes
7	EQ-5D-5L - Pain	The QOL-ACC Pain management dimension and EQ-5D-5L Pain/Discomfort are measuring similar constructs and therefore a positive and low-moderate correlation is expected.	Yes
8	EQ-5D-5L Anxiety/Depression	Self-reported happiness is found to be higher for those without any anxiety or depression symptoms and therefore a moderate to high correlation was expected between the QOL-ACC emotional wellbeing dimension and EQ-5D-5L Anxiety/Depression dimension [30].	Yes
9	EQ-5D-5L Self-care	A high correlation between the Independence dimension and EQ-5D-5L Self-care dimension was found in an aged care sample and similarly a moderate to high correlation was expected in our sample [13].	Yes
10	EQ-5D-5L Usual Activities	A moderate to high correlation was expected between the conceptually similar EQ-5D Usual Activities dimension and the QOL-ACC Activity dimension.	Yes
11	EQ-5D-5L Usual Activities	Correlation has been found between strong social connections and social capital and the participation in hobbies and leisure activities for older people therefore a low to moderate positive correlation was expected between the QOL-ACC Social Connection dimension and the EQ-5D-5L Usual Activities dimension [31].	Yes
12	UCSQ- Progress	It was expected that if respondents’ health issue had been resolved by their visit to the urgent or emergency care clinic that their pain levels would be lower than those for whom it was not resolved therefore a low to moderate correlation was expected between the QOL-ACC pain dimension and UCSQ Progress score.	Yes
13	UCSQ - Progress	It was expected that if respondents’ health issue had been resolved by their visit to the urgent or emergency care clinic and/or they were experiencing good quality, ongoing care then they would also be experiencing higher emotional wellbeing therefore a low to moderate correlation was expected between the QOL-ACC emotional wellbeing dimension and UCSQ Progress score.	Yes
**Known-group (4)**			
14	Care Needs at Home (binary)	Those respondents who have care needs at home are likely to have lower QOL-ACC utility scores	**NO**
15	Self-Rated Health	Those who report better health on the global self-rated health question are likely to have higher QOL-ACC utility scores	YES
16	Self-Rated QOL	Those who report better QOL on the global self-rated QOL question are likely to have higher QOL-ACC utility scores	YES
17	SEIFA-IRSAD	Those who reside in areas of more economic advantage according the SEIFA-IRSAD data are likely to have higher QOL-ACC utility scores [32].	**NO**

QOL-ACC – Quality of Life – Aged Care Consumers, UCSQ – Urgent Care Service Questionnaire, EQ-VAS – EuroQol Visual Analog Scale, EQ-5D-5L – EurQol 5 Dimensions – 5 level, SEIFA-IRSAD – Socio-Economic Indexes For Areas – Index of Relative Socio-economic Advantage and Disadvantage

### Feasibility

Feasibility was assessed by examining missing responses and ceiling effects. Ceiling effects arise when QOL-ACC index scores are predominantly concentrated at the upper limit of the scale (1.0). For this study, missing responses ≤ 5% and ceiling effects ≤ 15% were considered evidence of adequate feasibility.

### Statistical analysis

Data were analysed using SPSS statistical analysis software (Version 28.0.1.0). Descriptive statistics were generated for demographic information. Australian population scoring algorithms were applied to scores on both the QOL-ACC and the EQ-5D-5L to enable the generation of utility scores. Kruskal Wallis test was used to assess the difference between EQ-5D-5L, EQ-VAS and UCSQ Entry, UCSQ Convenience and UCSQ Progress scores at the QOL-ACC dimension level. For convergent validity correlation was assessed using Spearman’s Rank absolute correlation co-efficient (ρ). For known-group validity t-tests were performed for hypothesis 14 which included only two groups, whilst Kruskal Wallis was used to test for differences across groups for hypotheses 15, 16 and 17 (Table 1). Box plots were generated to visually demonstrate the differences between groups. Post-hoc multiple pairwise comparisons were performed using Dunn’s test [33]. Statistical significance was set at 0.05 for all tests.

## Results

### Sample

In total 625 potential respondents were contacted by Pure Profile researchers. Of these, 360 were excluded due to not meeting the eligibility criteria and a further 48 respondents met eligibility criteria but did not complete the surveys, representing a relatively high completion rate of 82% among eligible respondents [35]. A total of 12 respondents had attended an urgent/emergency care clinic as a companion and as such these were also excluded. Of the remaining 205 respondents, 59% (*n* = 121) were female and had a mean age of 75 (± 6). About one in five respondents were receiving formal aged care services (*n* = 37, 18.0%). Participants were slightly skewed across areas of socio-economic advantage with a higher proportion dwelling in areas of higher advantage according to the Socio-Economic Indexes for Areas (SEIFA) – Index of Relative Socio-economic Advantage and Disadvantage (IRSAD), Australia (2021 census data) [36]. Mean utility scores for all participants were higher for the EQ-5D-5L (0.82, ± 0.21) than for the QOL-ACC (0.73 ± 0.25) (Table 2).

**Table 2:** Socio-demographic characteristics of the respondents

Variables	*N* = 205 (100%)
Gender, *N* (%)	
Male	84 (41)
Female	121 (59)
Age, *N* (%)	
65–74	94 (45.9)
75–84	95 (46.3)
85+	16 (7.8)
Mean Age (SD)	75 (6.0)
Median Age (IQR)	75 (69.5–79)
Range	65–89
Care packages and levels, *N* (%)	
Not receiving formal care	159 (77.6)
Commonwealth Home Support Programme (CHSP)	10 (4.9)
Home Care Package – Level 1	9 (4.4)
Home Care Package – Level 2	11 (5.4)
Home Care Package – Level 3	3 (1.5)
Home Care Package – Level 4	4 (2)
Unsure	9 (4.4)
Informal Care, *N* (%)	
Informal carer	58 (28.3)
No informal carer	147 (71.7)
Living Arrangements, *N* (%)	
Living Alone	66 (32.2)
Living with spouse/partner	124 (60.5)
Living with other relatives	10 (4.9)
Living with other (s) – not relatives	4 (2)
Living in an aged care home	1 (0.5)
Country of Birth, *N* (%)	
Australia	156 (76.1)
Other	49 (23.9)
Highest Educational Qualification, *N* (%)	
No qualifications	10 (21.7)
Completed High School	9 (19.6)
Undergraduate degree or professional qualification	11 (23.9)
Post-graduate qualification	3 (6.5)
Other	4 (8.7)
SEIFA IRSEAD - quintiles, *N* (%)	
1 (least advantaged)	31 (15.1)
2	25 (12.2)
3	47 (22.9)
4	43 (21)
5 (most advantaged)	59 (28.8)
Self-reported health, *N* (%)	
Excellent	9 (4.4)
Very Good	48 (23.4)
Good	81 (39.5)
Fair	53 (25.9)
Poor	14 (6.8)
Self-reported quality-of-life, *N* (%)	
Excellent	13 (6.3)
Very Good	77 (37.6)
Good	70 (34.1)
Fair	36 (17.6)
Poor	9 (4.4)
EQ-5D-5L (mean ± SD)	0.82 ± 0.21
QOL-ACC (mean ± SD)	0.73 ± 0.25
UCSQ Entry (mean ± SD)	3.84 ± 0.74
UCSQ Convenience (mean ± SD)	3.46 ± 0.64
UCSQ Progress (mean ± SD)	3.8 ± 0.74

### Feasibility

There were no missing data with all 205 respondents completing the QOL-ACC in full. A minor ceiling effect (9.3%) was noted, supporting the feasibility of the QOL-ACC tool for use among older people accessing urgent and emergency care services.

### Construct validity

#### ***Convergent validity of the QOL-ACC***

The highest correlation for overall QOL-ACC utility scores was found with the EQ-5D-5L utility score (ρ = 0.60, *P* < 0.001, hypothesis 1). A similar level of correlation was demonstrated with the EQ-VAS (ρ = 0.57, *P* < 0.001, hypothesis 2). The QOL-ACC showed negligible correlation with UCSQ Entry scores (ρ = 0.27, *P* < 0.001, hypothesis 5) and relatively higher, but in the low-moderate range, correlation with UCSQ Convenience (ρ = 0.34, *P* < 0.001, hypothesis 4). As expected, of the UCSQ dimensions, highest correlation was demonstrated with the progress score (ρ = 0.37, *P* < 0.001, hypothesis 5) (Table 2).

#### Mobility

The Mobility dimension demonstrated the highest correlation with the EQ-5D-5L utility score (ρ = 0.43, *P* = < 0.001) and the EQ-5D-5L dimension of Mobility (ρ = 0.41, *P* = < 0.001, hypothesis 6). There was only marginal correlation with the EQ-5D-5L dimension of Anxiety/Depression (ρ = 0.17, *P* = 0.014) (Table 3).

**Table 3:** Relationship between the QOL-ACC and other instruments (Construct Validity)

	QOL-ACC, Spearman’s rho correlation co-efficient (*P* values)						
	Overall Score	Mobility	Pain	Independence	Emotional well-being	Social Connection	Activity
EQ-5D-5L	0.60(< 0.001)	0.43(< 0.001)	0.32(< 0.001)	0.60(< 0.001)	0.46(< 0.001)	0.39(< 0.001)	0.42(< 0.001)
Mobility	0.46(< 0.001)	0.41(< 0.001)	0.22(0.002)	0.53(< 0.001)	0.32(< 0.001)	0.24(< 0.001)	0.35(< 0.001)
Self-care	0.36(< 0.001)	0.40(< 0.001)	**0.14(0.53)**	0.46(< 0.001)	0.17(0.02)	0.22(0.002)	0.22(0.002)
Usual Activities	0.52(< 0.001)	0.40(< 0.001)	0.32(< 0.001)	0.55(< 0.001)	0.35(< 0.001)	0.32(< 0.001)	0.36(< 0.001)
Pain and Discomfort	0.49(< 0.001)	0.32(< 0.001)	0.44(< 0.001)	0.43(< 0.001)	0.39(< 0.001)	0.31(< 0.001)	0.32(< 0.001)
Anxiety and Depression	0.35(< 0.001)	0.17(0.014)	**0.13(0.06)**	0.28(< 0.001)	0.40(< 0.001)	0.31(< 0.001)	0.22(< 0.001)
EQ-5D VAS	0.57(< 0.001)	0.38(< 0.001)	0.36(< 0.001)	0.52(< 0.001)	0.45(< 0.001)	0.35(< 0.001)	0.45(< 0.001)
*UCSQ*							
Entry	0.27(< 0.001)	**0.12(0.07**)	0.29(< 0.001)	0.21(0.003)	0.24(< 0.001)	0.21(0.001)	**0.10(0.14)**
Convenience	0.34(< 0.001)	**0.15(0.30)**	0.45(< 0.001)	0.22(0.002)	0.32(< 0.001)	0.15(0.038)	0.21(0.003)
Progress	0.37(< 0.001)	0.17(0.014)	0.40(< 0.001)	0.29(< 0.001)	0.36(< 0.001)	0.20(0.004)	0.21(0.003)
Health – Global Question	55(< 0.001)	0.36(< 0.001)	0.38(< 0.001)	0.49(< 0.001)	0.46(< 0.001)	0.36(< 0.001)	0.47(< 0.001)
QOL – Global Question	0.58(< 0.001)	0.38(< 0.001)	0.38(< 0.001)	0.51(< 0.001)	0.48(< 0.001)	0.41(< 0.001)	0.46(< 0.001)

#### Pain Management

The Pain Management dimension had highest correlation with the UCSQ convenience dimension (ρ = 0.45, *P* = < 0.001). It was also moderately correlated with the EQ-5D-5L Pain/Discomfort dimension (ρ = 0.44, *P* = < 0.001, hypothesis 7). It demonstrated similar moderate correlation with the QOL and health global questions (ρ = 0.38, *P* = < 0.001) as well as the EQ VAS (ρ = 0.36, *P* = < 0.001) (Table 3).

#### Independence

The QOL-ACC dimension of Independence was highly correlated with the EQ-5D-5L utility score (ρ = 0.60, *P* = < 0.001) as well as the QOL-ACC dimension of Usual Activities (ρ = 0.55, *P* = < 0.001). It also showed moderate correlation with the EQ-5D-5L dimension of Mobility (ρ = 0.53, *P* = < 0.001), Self-care (ρ = 0.46, *P* = < 0.001, hypothesis 9) and Pain/Discomfort (ρ = 0.43, *P* = < 0.001). The Independence dimension was more highly correlated with the global QOL question (ρ = 0.51, *P* = < 0.001) than the global health question (ρ = 0.49, *P* = < 0.001) (Table 3).

#### Social Connection

The Social Connection dimension demonstrated the highest correlation with the QOL global question (ρ = 0.41, *P* = < 0.001) and the EQ-5D-5L utility score (ρ = 0.39, *P* = < 0.001). On a dimension level, the highest correlation found with the Usual Activities dimension (ρ = 0.32, *P* = < 0.001, hypothesis 11). Moderate correlation was also demonstrated with the Pain/Discomfort and Anxiety/Depression dimensions (ρ = 0.31, *P* = < 0.001) (Table 3).

#### Emotional Wellbeing

The Emotional Wellbeing dimension demonstrated moderate correlation with the QOL global question (ρ = 0.48, *P* = < 0.001), and the global the health questions (ρ = 0.46, *P* = < 0.001) as well as the EQ-5D-5L utility scores (ρ = 0.46, *P* = < 0.001) and EQ-VAS (ρ = 0.45, *P* = < 0.001). It also showed moderate correlation with the UCSQ progress score (ρ = 0.36, *P* = < 0.001). On a dimension level, the highest correlation was with the Anxiety/Depression (ρ = 0.40, *P* = < 0.001, hypothesis 8) and Pain/Discomfort (ρ = 0.39, *P* = < 0.001) dimensions of the EQ-5D-5L (Table 3).

#### Activity

The Activity dimension showed higher correlation with global health question (ρ = 0.47, *P* = < 0.001) than with the global QOL question (ρ = 0.46, *P* = < 0.001) Moderate correlation was demonstrated with the EQ-5D Usual Activities (ρ = 0.36, *P* = < 0.001, hypothesis 10) and Mobility (ρ = 0.35, *P* = < 0.001) dimensions (Table 3).

#### Known-group validity

**Fig. 1:**
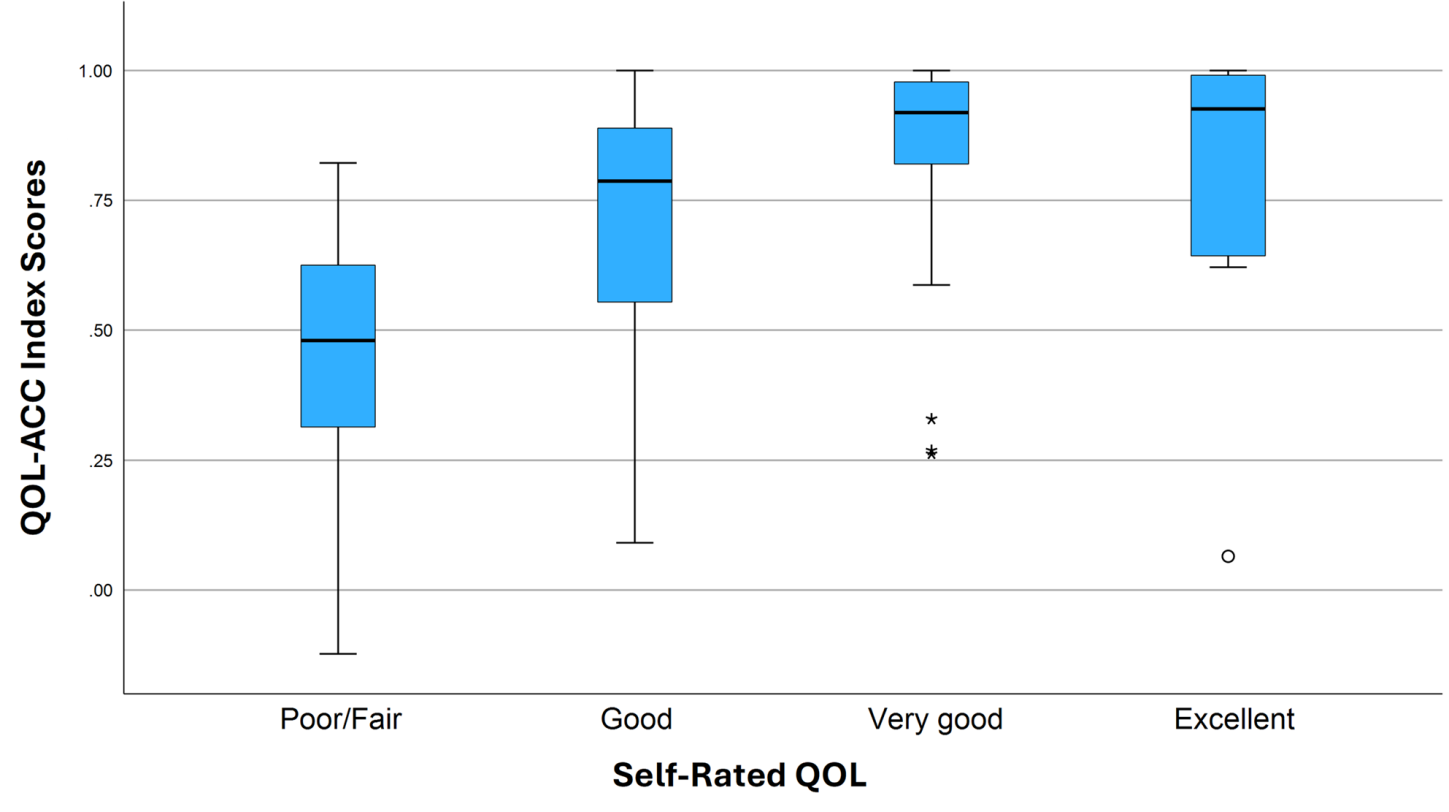
Box plot of QOL-ACC utility scores across levels of self-rated QOL. The box represents the interquartile range (IQR), with the median shown as a horizontal line inside the box. Whiskers extend to values within 1.5 × IQR from the quartiles. Mild outliers (1.5–3 × IQR) are indicated with small circles, and extreme outliers (> 3 × IQR) with asterisks

**Fig. 2:**
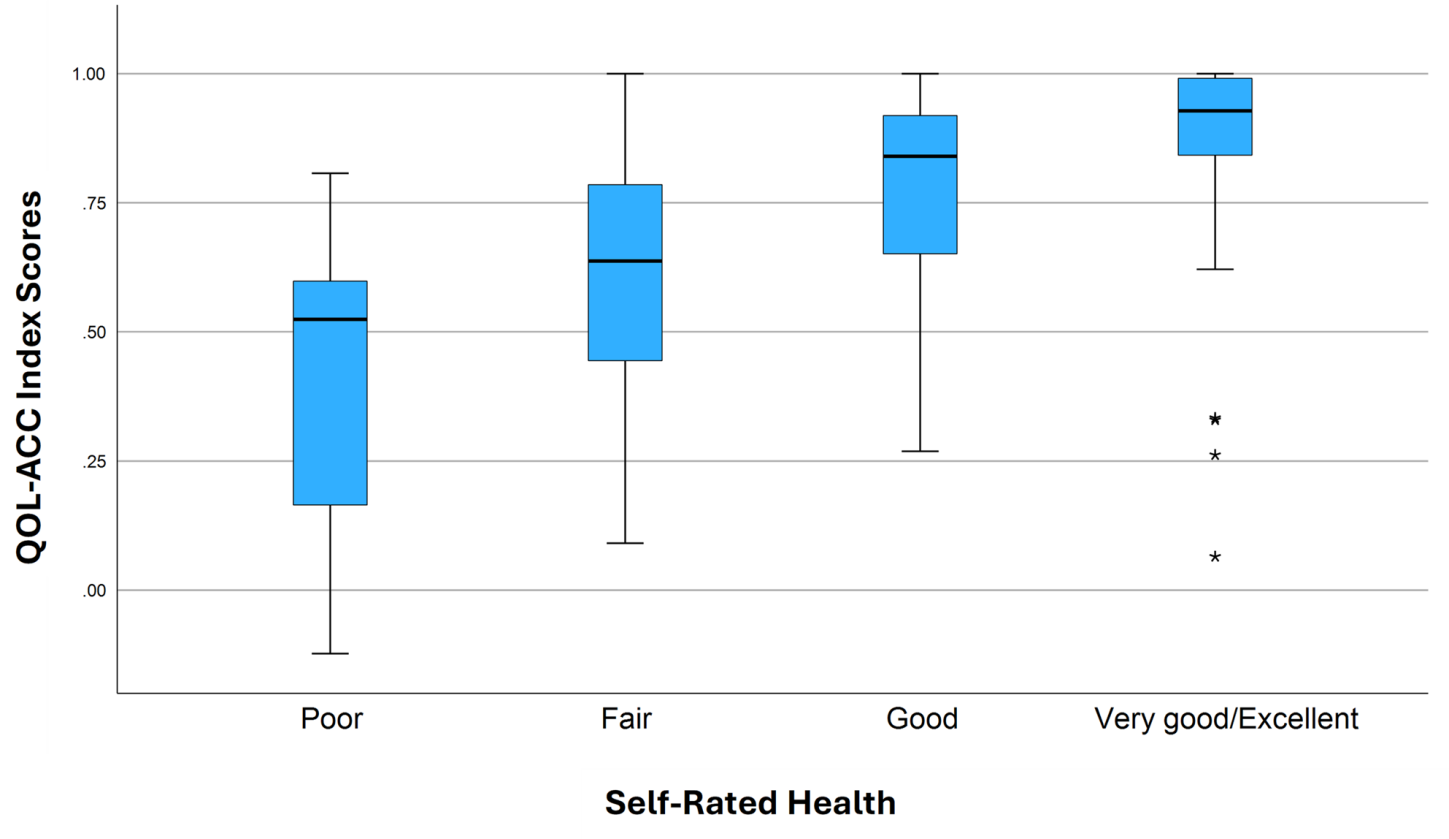
Box plot of QOL-ACC utility scores across levels of self-rated health. The box represents the interquartile range (IQR), with the median shown as a horizontal line inside the box. Whiskers extend to values within 1.5 × IQR from the quartiles. Extreme outliers (> 3 × IQR) are indicated with asterisks

**Fig. 3:**
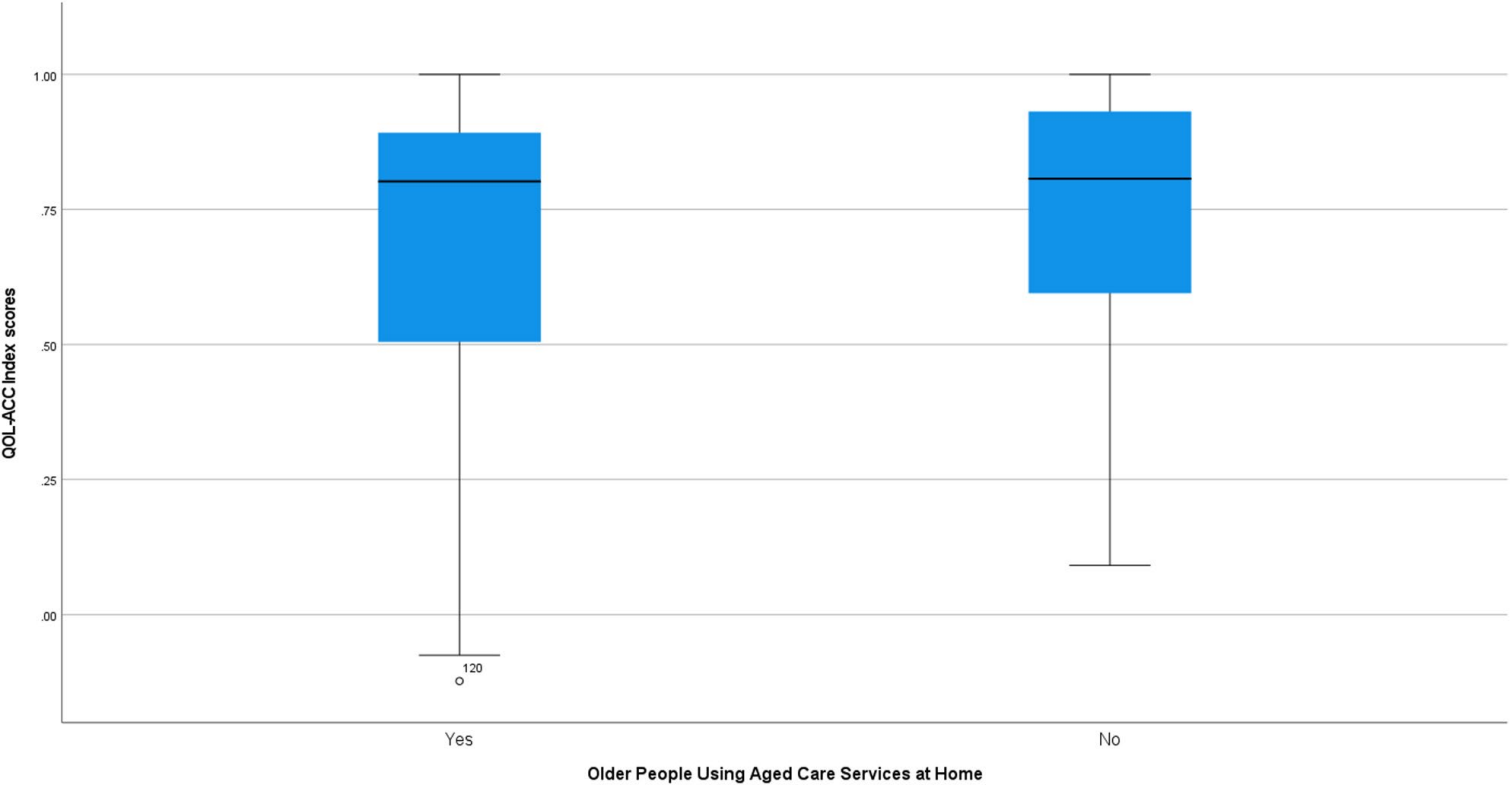
Box plot of QOL-ACC utility scores by age care service use. The box represents the interquartile range (IQR), with the median shown as a horizontal line inside the box. Whiskers extend to values within 1.5 × IQR from the quartiles. Mild outlier (1.5–3 × IQR) is indicated with a small circle

The QOL-ACC utility scores discriminated across respondents with varying levels of self-rated QOL (Fig. 1) and self-rated health (Fig. 2). While those receiving aged care services at home demonstrated lower scores on the QOL-ACC (Fig. 3), this difference was not significant (*P* = 0.06, hypothesis 14). There was no statistically significant difference in utility scores by area of lower relative socio-economic advantage (hypothesis 17). Statistically significant differences in QOL-ACC utility scores by self-rated levels of QOL were demonstrated (*P* < 0.001) (hypothesis 16). Kruskal-Wallis H tests also showed statistically significant differences between utility scores of participants with different levels of self-rated health (*P* < 0.001) (hypothesis 15). (See additional file 1 for post-hoc pairwise comparisons.)

Overall, 14 of the 17 convergent and known group validity hypotheses (82%) were confirmed suggesting that the QOL-ACC utility scores and descriptive system have demonstrated satisfactory evidence of construct validity (Table 1).

## Discussion

This study provides strong evidence for the construct validity of the QOL-ACC at both dimension level and overall, for use with older community dwelling urgent and emergency care services users. To our best knowledge, this is the first study in Australia and internationally to assess the construct validity of the QOL-ACC tool within a health system user sample. This study found that the mean utility score derived from the QOL-ACC (0.73) was lower than that estimated using the EQ-5D-5L (0.82) in a sample of community-dwelling older people. In contrast, studies conducted in residential aged care settings reported lower mean utilities on both of these measures (ranging from 0.57 to 0.74), reflecting the greater health and social care needs of individuals in these settings [17, 18]. The relatively lower QOL-ACC score observed in this study may indicate that the instrument is more sensitive to aspects of QOL that are less apparent in the EQ-5D-5L, particularly in a community dwelling sample of older people. Unlike the EQ-5D-5L, the QOL-ACC captures a broader range of psychosocial dimensions, which may provide a more comprehensive assessment of QOL in this group.

Correlations with UCSQ dimensions were low to moderate. As hypothesised, the relationship between with the QOL-ACC utilities and UCSQ Progress dimension was stronger than with the Convenience and Entry dimensions. This was also consistent with the findings of Khadka et al. [18] and Hutchinson et al. [17] demonstrating a strong association between quality of care and QOL in a residential aged care sample who received 24-hour daily care and for whom the care experience and clinical outcomes were integral to their daily life. The current study extends this finding and indicates that even in a sample not receiving ongoing care, the quality of care for older people was slightly but significantly correlated with QOL when assessed using the QOL-ACC. Khadka et al. found a similar moderate correlation with the Quality of Care Experience (QCE) measure indicating that those receiving aged care at home resemble the community-dwelling older people included in this study [37]. Although quality of care and QOL are conceptually distinct, the observed associations support the convergent validity of the QOL-ACC in capturing dimensions of older people’s experiences in relation to care quality.

The Emotional Wellbeing dimension of the QOL-ACC showed moderate correlation with the Pain/Discomfort dimension of the EQ-5D-5L, an expected relationship given previous findings on the association between Pain and Emotional Wellbeing [38, 39]. Notably however, the Pain Management dimension of the QOL-ACC showed no statistically significant correlation with the Anxiety/Depression dimension of the EQ-5D-5L. This finding may indicate that while the Pain Management and Emotional Wellbeing dimensions of the QOL-ACC are similar to the Pain/Discomfort dimension and Anxiety/Depression dimensions of the EQ-5D-5L, respectively, they are, in fact, measuring distinct constructs. This may suggest that the QOL-ACC dimensions capture broader aspects of QOL in older people such as pain management and happiness rather than the health focussed physical pain/discomfort or anxiety/depression states measured by the EQ-5D-5L. Additionally, composite dimensions have been found to be challenging for respondents to interpret and this may have been a contributing factor in this finding [40]. A recent study by Khanna et al. in a sample of children aged 6–10 years found that children sometimes conflated Pain/Discomfort of the EQ-5D-Y-3L with emotional pain [41]. Additionally, a sample of older people in aged care found issues with the combination of health states in the Anxiety/Depression dimension [42]. Most dimensions of the QOL-ACC demonstrated a stronger correlation with the global self-report QOL question than the health question further indicating the QOL as measured by the QOL-ACC is broader and distinct from HRQOL as measured by the EQ-5D-5L. The only dimension of the QOL-ACC to demonstrate stronger correlation with the global self-report health question than with the global self-report QOL question was the Activity dimension. This may represent the impact of poor health on the ability or willingness to participate in activities, drivers of which can include pain or perceived risk of injury [43]. It may also reflect the mediating effect that participation in leisure activities can have on health outcomes for older people [44]. Overall, these results inform the construct validity of the QOL-ACC as a complementary measure that addresses QOL dimensions particularly relevant to older people receiving or anticipating care.

The QOL-ACC demonstrated discriminate validity between respondents with different levels of self-reported health and self-reported QOL. However, differences between QOL-ACC scores based on frequency of urgent/emergency care visits was insignificant. It is worth noting that people who had received urgent or emergency care twice or more in the previous year did report lower QOL on the QOL-ACC, but it was not statistically significant, this was likely due to the low power of the sample for the more frequent urgent/emergency care user cohort. For the SIEFA-IRSAD groups distribution of utility scores across SEIFA-IRSAD quintiles showed that while the difference was not statistically significant, those in the quintile 1 (the area of lowest relative advantage) and those in quintiles 4 and 5 (areas of highest relative advantage) demonstrated higher QOL-ACC utility scores than those in the quintiles 2 and 3. This finding is somewhat consistent with the literature which suggests a link between socio-economic advantage and QOL, however, the dynamic is somewhat complicated [45–47]. For example, in a large-scale study of Australian men, those living in high and low socio-economic areas were found to have lower QOL overall than those in the middle quadrants [48], the opposite of the finding in the current study.

This study has several limitations which are important to highlight. Surveys were completed online and as such there may have been some response bias given that only older people with access and the ability to complete surveys online could be included. This may have caused an over-representation of people with higher education and an under-representation of people with cultural and linguistically diverse backgrounds. Additionally, our sample included an overrepresentation of female respondents and younger age groups (aged 65–84) when compared to the national patterns of emergency department presentations [3]. However, the study sample included relatively even distribution amongst areas of socio-economic advantage/disadvantage. The cognitive status of this sample was not assessed, and the recall may not be accurate for some older adults in this cohort. However, previous work by Lay et al. [21] has suggested that older people with mild to moderate cognitive impairment are able to reliably and independently self-complete the QOL-ACC and the levels of cognitive impairment are likely to be lower in this community dwelling sample. Research is currently underway to develop an easy-read version of the QOL-ACC which may further facilitate the use of the QOL-ACC with this cohort. Additionally older people accessing urgent or emergency care may not always have the capacity to self-complete. In this context proxy completion may be more appropriate. Proxy versions of the QOL-ACC are available and validated in long-term aged care settings, however the validity of the proxy versions in health settings remains to be established.

## Conclusion

This study provides construct validity evidence for the QOL-ACC tool and its associated dimensions for the assessment of the QOL of older urgent/emergency care users living in the community. The QOL-ACC, developed from inception in partnership with older people, is now utlised nationally for the quality assessment of residential aged care services and shows promise for use with older people receiving home care and health services. In the context of urgent care, the QOL-ACC holds similar potential for application in evaluation, quality improvement, and comparative effectiveness research, given that many older people who use aged care services also access urgent care frequently. This study represents the first step in validating this instrument in this setting, with the longer-term goal of enabling its integration to support clinical conversations, care planning and benchmarking service quality.

## Supplementary Information

Below is the link to the electronic supplementary material.

Supplementary Material 1

## Data Availability

Data are not publicly available due to Flinders University ethics requirements but are available from the corresponding author on reasonable request.

## Abbreviations

QOL-ACC: Quality-of-life – aged care consumers
EQ-5D-5L: EuroQoL 5 Dimensions – 5 level
EQ-VAS: EuroQoL Visual Analog Scale
UCSQ: Urgent Care Service Questionnaire
SEIFA-IRSAD: Socio-Economic Indexes For Areas – Index of Relative Socio-economic Advantage and Disadvantage
QALY: Quality Adjusted Life Year
HRQOL: Health-Related Quality-of-life
QOL: Quality-of-life
PBM: Preference-Based Measure
QCE: Quality of Care Experience
COSMIN: Consensus-based Standards for the selection of health Measurement INstruments
