# Supplementary material for: Validation of a quality-of-life measure for older people using urgent and emergency care
Source: Health Qual Life Outcomes. 2025 Nov 28;24:2. doi: 10.1186/s12955-025-02454-z (PMC12764127; doi:10.1186/s12955-025-02454-z)
Supplement: Supplementary file 1 — Supplementary Material 1 [file 12955_2025_2454_MOESM1_ESM.pdf]

Validation of a Quality-of-Life measure for older people using urgent and emergency care.

Health and Quality of Life Outcomes

Kiri Lay[1], Julie Ratcliffe[1], Rachel Milte[1], Diana Khanna[1], Craig Whitehead[2], Jyoti Khadka[1]

[1]Health and Social Care Economics Group, Caring Futures Institute, Flinders University, Australia

[2]Rehabilitation, Aged and Palliative Care, Southern Adelaide Local Health Network, South Australia, Australia

Corresponding Author: Kiri Lay, Health and Social Care Economics Group, Caring Futures Institute, Flinders University, Adelaide, GPO Box 2100, Adelaide, SA 5001, Australia. E: kiri.lay@flinders.edu.au

Known group post-hoc pairwise comparisons.

	Test Statistic	*P*	Adj Sig.
Self-Rated QOL			
Poor/Fair - Good	-51.24	<0.001	.000
Poor/Fair - Excellent	-80.40	<0.001	.000
Poor/Fair – Very Good	-94.71	<0.001	.000
**Good - Excellent**	**-29.16**	**.103**	**.621**
Good – Very good	-43.47	<0.001	.000
**Excellent – Very good**	**14.31**	**.421**	**1.00**
Self-Rated Health			
**Poor - Fair**	**-1.62**	**.106**	**.636**
Poor - Good	-3.98	<0.001	.000
Poor – Very Good/Excellent	-5.90	<0.001	.000
Fair - Good	-3.77	<0.001	.001
Fair – Very Good/Excellent	-6.68	<0.001	.000
Good – Very Good/Excellent	-3.53	<0.001	.003
IRSEA – Areas of relative economic disadvantage			
**2-3**	**-4.72**	**.75**	**1.00**
**2-1**	**13.50**	**.40**	**1.00**
**2-5**	**-16.89**	**.23**	**1.00**
**2-4**	**-35.06**	**.20**	**.187**
**3-1**	**8.78**	**.52**	**1.00**
**3-5**	**-12.17**	**.29**	**1.00**
**3-4**	**-30.34**	**.02**	**.153**
**1-5**	**-3.40**	**.80**	**1.00**
**1.4**	**-21.56**	**.12**	**1.00**
**5-4**	**18.17**	**.13**	**1.00**
